# Long-term outcome after treatment of large uveal melanoma

**DOI:** 10.1007/s10792-025-03624-0

**Published:** 2025-07-08

**Authors:** Leyla Jabbarli, Mael Lever, Tobias Kiefer, Eva Biewald, Philipp Rating, Maja Guberina, Dirk Flühs, Nika Guberina, Ramazan Jabbarli, Martin Stuschke, Nikolaos E. Bechrakis, Theodora Tsimpaki, Miltiadis Fiorentzis

**Affiliations:** 1 Department of Ophthalmology, University Hospital Essen, Essen, Germany; 2 Department of Radiotherapy, University Hospital Essen, Essen, Germany; 3 German Cancer Consortium (DKTK), Heidelberg, Partner Site University Hospital Essen, Essen, Germany; 4 Department of Neurosurgery, University Hospital Essen, Essen, Germany

**Keywords:** Large uveal melanoma, Endoresection, Brachytherapy, Local recurrence, Enucleation

## Abstract

**Purpose:**

The management of large uveal melanomas (UM) remains a significant challenge. Treatment options include radiotherapy alone or in combination with surgical interventions. This study evaluates the long-term outcomes of patients with large UM, focusing on the impact of different treatment strategies.

**Methods:**

All consecutive patients with large UM treated at our institution primarily with an eye-salvaging approach—either bi-nuclide plaque brachytherapy (BNPB) or endoresection (ER) following neoadjuvant irradiation—between January 2014 and December 2020 were included in this study. Tumors with a thickness greater than 7.0 mm that were not suitable for ruthenium-106 plaque brachytherapy were classified as large. The primary study endpoints were the incidence of legal blindness of the affected eye at 12 and 36 months, as well as eye preservation and local tumor control at 36 months.

**Results:**

Overall, 194 patients underwent ER, while 204 received BNPB. At 12 and 36 months, legal blindness was documented in 25.5% and 39.8% of patients, respectively. Three years after treatment, eye preservation was achieved in 88.0% of cases, and local tumor control was observed in 92.9%. Multivariable analysis revealed a significantly lower risk of legal blindness at 12 months and secondary enucleation at 36 months in patients treated with ER without adjuvant brachytherapy.

**Conclusions:**

Large UM can be effectively managed, achieving both anatomical and functional eye preservation for several years post-treatment. Among the approaches investigated, ER following neoadjuvant irradiation demonstrated the most favorable long-term outcomes, with a lower incidence of legal blindness and secondary enucleation.

**Supplementary Information:**

The online version contains supplementary material available at 10.1007/s10792-025-03624-0.

## Introduction

Eye retaining therapy of uveal melanoma (UM) is currently standard of care not only for medium-sized, but also for large UM up to 11–12 mm in tumor thickness. Despite several therapeutic approaches, the choice of the optimal treatment modality for large tumors remains challenging, due to the expected radiogenic side-effects and the potential for development of a toxic-tumor syndrome [1, 2]. The most common globe-sparing options include episcleral brachytherapy and proton beam radiotherapy [3]. Episcleral brachytherapy for large UMs is commonly performed using ^125^I, which enables effective irradiation of large tumors due to deeper tissue penetration [4].

Some specialized centers offer also radiotherapy combined with surgical tumor resection (STR) in selected cases [2, 5, 6]. Tumors, with a basal diameter < 20 mm that are not feasible to brachytherapy can be treated with this combined therapy method, consisting of STR with neoadjuvant or adjuvant irradiation. STR not just eliminates the irradiated tumor tissue but also enables access to histopathologic and cytogenetic data [2]. Depending on tumor location, there are two alternative surgical approaches: tumors with a more anterior location and ciliary body involvement can be resected transsclerally via an external approach, while posterior UM’s can be removed via an internal transvitreal/transretinal approach. The latter was introduced in 1986 [7] and was originally considered as a beneficial treatment for juxtapapillary melanoma, avoiding radiation damage to the fovea and optic nerve [5].

Surgical techniques have evolved over time, helping to improve anatomical and functional outcomes and have become a valuable alternative therapy approach for large UM in certain cases [2, 5, 8, 9].

Differences in the availability of specific treatment modalities, the lack of comparative studies and conflicting findings in existing research offer a space for further investigation in this field.

Reports on the outcomes of treatment for large UMs are limited, and those comparing results across different modalities have yielded controversial findings [10–13]. Furthermore, there is a lack of uniformly collected long-term treatment outcomes, making it difficult to compare patient results at identical time intervals across different therapeutic approaches. To address this gap, we initiated this long-term study to compare various treatment modalities in patients with large UM.

In our specialized ophthalmic oncologic institution, we treat large UM with both episcleral brachytherapy and STR. Moreover, to enhance local tumor control while minimizing post-radiation toxicity, we developed a novel approach with bi-nuclide radioactive plaques, combining radiation properties ^106^Ru and ^125^I for optimized therapeutic efficacy [4].

In the present study, we aimed to evaluate the long-term functional outcomes, tumor control, and eye preservation rates following the treatment of large UM in a comprehensive, single-center cohort with special emphasis on assessing the potential impact of different treatment strategies on clinical outcomes.

## Methods

### Patient population

This retrospective study is based on the institutional database containing all consecutive patients with UM and tumor thickness exceeding 7 mm treated with eye preserving therapy consisting of bi-nuclide plaque brachytherapy (BNPB) or endoresection (ER) at the Departments of Ophthalmology and Radiotherapy at University Hospital Essen between January 1, 2014 and December 31, 2020. The study was approved by Institutional Ethics Committee (Ethik-Kommission, Medizinische Fakultät der Universität Duisburg-Essen, registration number 20–9165-BO). Patients’ electronic medical records were reviewed until November 2023.

### UM management

UM was diagnosed clinically. Colored fundus photos, ultrasonography or/and ultrasound biomicroscopy was performed at initial examination and during follow-ups for tumor documentation. In certain cases, a transillumination was performed at initial diagnosis to verify a ciliary body involvement. The choice of therapy modality depended on largest tumor thickness (LTT) and largest tumor basal diameter (LTBD), assessed with ultrasonography prior the therapy as well as the location of tumor regarding neighbor structures (iris and/or ciliary body involvement by anterior tumors, proximity to optic nerve by posterior tumors).

Tumors with thickness > 11.0 mm and LTBD > 20.0 mm underwent primary enucleation. UM with thickness > 7.0 mm and ≤ 11.0 mm were managed with bi-nuclide radioactive plaques, consisting of ^125^I seeds glued at silicone inlay in the inner concave surface of a regular 20 mm ^106^Ru plaque CCB type embedded in a golden plaque. Detailed description of bi-nuclide radioactive plaques was published previously [4, 14]. The prescribed minimum dose for the tumor apex was 84 Gy for tumors ≤ 8.0 mm in height and 70 Gy for tumors > 8.0 mm, whereby the dose for the sclera was at least 700 Gy but did not exceed 1500 Gy.

STR was indicated by tumor thickness > 7.0 mm and LTBD < 20.0 mm. Anterior tumors with ciliary body involvement were managed with transscleral resection under systemic hypotension and hypothermia if no contraindications for anesthesia were estimated. Posterior UM with minimal proximity of 3 mm to optic nerve were treated with pars plana vitrectomy and transretinal ER. All patients eligible for STR underwent neoadjuvant irradiation administered in a single session under retrobulbar anesthesia. The neoadjuvant treatment consisted of Gamma Knife or stereotactic radiosurgery (SRS) using image-guided radiotherapy/volumetric modulated arc therapy (IGRT/VMAT) delivered via the TrueBeam/Novalis systems, applying to the tumor a minimum dose of 25 Gy by Gamma Knife and 22 Gy by SRS, respectively. In one case, a neoadjuvant brachytherapy was performed.

Within ten days after neoadjuvant irradiation, 25 gauge 4 port pars plana vitrectomy under general anesthesia was performed. Phacoemulsification with intraocular lens implantation prior to the pars plana vitrectomy was performed in phakic patients. Complete posterior vitreous detachment was induced. Transretinal tumor resection was performed with vitreous cutter, starting on tumor apex down to bare sclera. To minimize bleeding, the intraocular pressure was raised up to 80 mm Hg and the systolic blood pressure was reduced ≤ 100 mm Hg. After tumor resection, perfluorodecalin liquid was injected and laser retinopexy was performed along the edges of the retinectomy and choroidectomy. Subsequently, perfluorodecalin liquid was exchanged for silicon oil 5000 cSt. Patients treated up to November 2018 underwent adjuvant brachytherapy with ^106^Ru plaque in addition to neoadjuvant radiotherapy according to an internal agreement between the Ophthalmology and Radiotherapy Departments of the University Hospital Essen. A ^106^Ru plaque was applied directly after the ER. The prescription point for plaque dosimetry was defined in all cases as the apex of an imaginary 3.0-mm thick tumor residue receiving 100 Gy.

The standard postoperative treatment regime after brachytherapy was the application of combined steroid/antibacterial ointment (0.30 mg dexamethasone, 5 mg gentamicin sulphate) and tropicamide eye drops 3 times daily for 3 weeks with gradual reduction of the local medication. Application frequency of steroid and antibacterial eye drops during the postoperative treatment after ER was adapted depending on the severity of postoperative intraocular inflammation (up to hourly). This regime was constant throughout the study period.

**Fig. 1:**
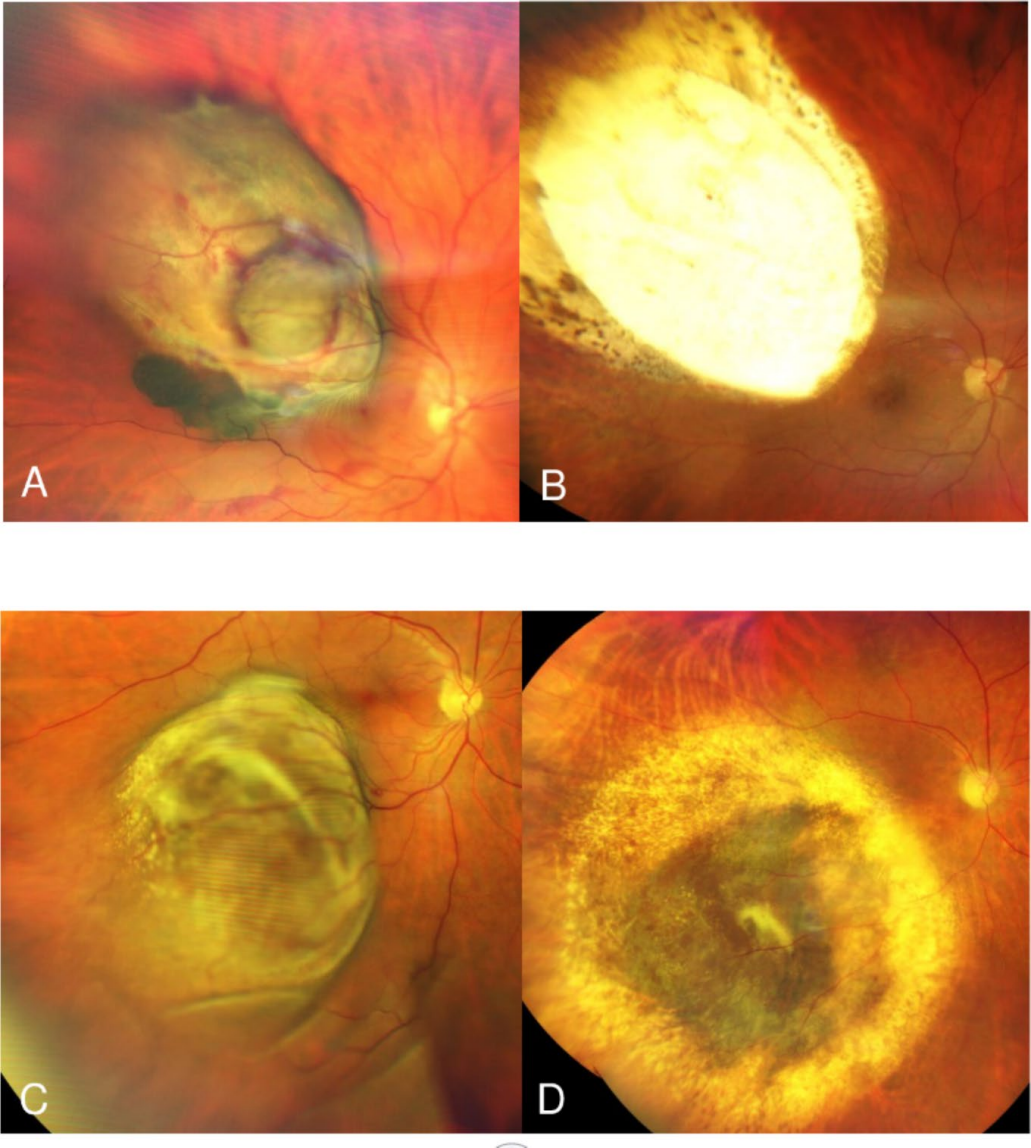
**A** Pre-treatment photo of a 69 year-old patient with uveal melanoma; **B** Same patient, 65 months after endoresection without adjuvant brachytherapy. **C** Pre-treatment photo of a 67 year-old patient with uveal melanoma; **D** Same patient, 46 months after bi-nuclide plaque brachytherapy

Follow-up examinations after BNPB were initially performed at 3 months intervals. The first follow-up examination after ER was scheduled to be in 4–6 weeks after the surgery. In stable cases, the follow-up intervals were extended up to 6, 9 and 12 months (Fig. 1). The patients with detected complications or side effects were monitored closely. Silicone oil was removed 6–9 months after ER.

### Data management

The documented tumor parameters were LTT and LBTD prior to the therapy (based on ultrasonography), location of posterior tumor margin (parapapillary, posterior to equator, anterior to equator), ciliary body involvement, extraocular extension, TNM-classification. UMs with posterior tumor margin within 5 mm proximity to optic nerve were defined as parapapillary tumors. Additionally, the following parameters were collected: patients’ age and sex, neoadjuvant irradiation prior to ER (gamma knife or stereotactic irradiation), time and indications of surgical interventions, scleral contact dose (SCD) and apex dose (AD), VA prior to surgery, at 12 and 36 months post-treatment, local recurrence (LR) and secondary enucleation (SE) at 36 months.

VA between 1.0 and 0.05 decimal was estimated on a decimal scale chart at a distance of 5 m. VA between 0.04 and 0.02 decimal was measured with a VA chart at a distance of 1 m. VA worse than 0.02 decimal was recorded as counting fingers at 1 m, hand movement, light perception and no light perception. VA data were converted to LogMAR units (logarithm of the minimum angular resolution) for further statistical evaluation. Legal blindness (LB) was defined as a VA of the affected eye of less than 0.05 in decimal notation.

In the VA analysis, patients who underwent secondary enucleation (SE) were classified as cases with complete vision loss at the time of SE. For the analysis of recurrence rates, patients who underwent SE without prior tumor recurrence were classified as lost to follow-up after SE.

### Statistical analysis

The goal of this study was to analyze the long-term functional and anatomical outcomes of therapy for UM lesions exceeding 7 mm in thickness and to assess specific characteristics based on the treatment modality. The primary study endpoint was the development of LB at 12 and 36 months after initial treatment. The secondary study endpoints were tumor control and eye preservation at 36 months follow-up.

According to the treatment strategies performed in the cohort, patients were first divided into three major groups: BNPB, ER with adjuvant brachytherapy (aBT), and ER without aBT. Differences between the treatment groups were assessed using univariable analysis. Continuous variables were analyzed using the Student t test for normally distributed data and the Mann–Whitney U test for non-normal distributed data. Associations between categorical variables were analyzed using the Chi-square or Fisher exact tests, as appropriate.

The associations between the baseline parameters and the study endpoints were evaluated in a multivariable binary logistic regression model with a backward stepwise conditional approach, where the group with the best treatment results was analyzed against the remaining cohort. Results were presented as the adjusted odds ratio with 95% confidence intervals. A *P* value < 0.05 was considered statistically significant.

Data analysis was performed with the use of SPSS (version 24, SPSS Inc., IBM, Chicago, IL, USA) statistical software.

## Results

Between January 2014 and December 2020, a total of 398 patients with UM and a tumor thickness > 7.0 mm received eye-preserving therapy with BNPB or ER at the Department of Ophthalmology and Radiotherapy of the University Hospital Essen and were included in the final analysis. 204 patients underwent BNPB and 194 were treated with ER. SRS using IGRT/VMAT was performed in 15 cases, and 178 patients received radiosurgery with the Gamma Knife. Of 194 ER patients, 127 underwent aBT with a ^106^Ru plaque, with a mean AD of 152.5 Gy and a mean SCD of 451.5 Gy being applied.

The main patients, tumor and radiation characteristics of the whole cohort are presented in Table 1.

**Table 1:** The main patients’, tumor and radiation characteristics of the analyzed cohort

Parameter	Number (percentage) or median (IQR)
Age, years	62.12 (52.58–72.38)
Sex (female)	181 (45.5%)
Treatment modality	
ER with aBT	127 (31.9%)
ER without aBT	67 (16.8%)
BNPB	204 (51.3%)
TNM	
T2a	63 (15.8%)
T2b	15 (3.8%)
T3a	190 (47.7%)
T3b	92 (23.1%)
T3c	3 (0.8%)
T3d	5 (1.3%)
T4a	18 (4.5%)
T4b	12 (3.0%)
Apex dose (Brachytherapy), Gy	85.5 (71.0–137.6)
Sclera contact dose (Brachytherapy), Gy	742.6 (445.8–944.9)
Extraocular extension	8 (2.0%)
Ciliary body involvement	128 (32.2%)
LTT, mm	8.77 (7.93–9.93)
Largest basal tumor diameter, mm	13.40 (11.46–15.57)
Posterior tumor margin*	
Parapapillary tumor location	119 (30.1%)
Posterior to equator	168 (42.4%)
Anterior to equator	106 (26.7%)
VA at diagnosis, decimal	0.4 (0.8–0.2)
Patients with LB at diagnosis	39 (9.8%)

BNPB, Bi-nuclide plaque brachytherapy; ER, Endoresection; aBT, Adjuvant brachytherapy; VA, Visual acuity; LB, Legal blindness; LTT, Largest tumor thickness

^*^In 5 cases posterior tumor margin was not documented

### Comparison between the treatment groups

The univariable analysis (Table 2) revealed that the tumors in the BNPB-group were broader (vs. ER with aBT**: *p* < 0.0001**; r = 0.449; vs. ER without aBT: ***p* < 0.0001**; r = 0.456), predominantly located more anteriorly, and fittingly showed significantly more frequent ciliary body involvement. In contrast, tumors in the ER-groups were primarily parapapillary (ER with aBT vs BBNP**:* p* < 0.0001**; 40% vs 17.7%); ER without aBT vs BNPB: ***p* < 0.0001**; 49.3% vs 17.7%). The median VA at baseline was in the entire cohort 0.4 decimal (interquartile range (IQR) 0.8–0.2) and was comparable across all three treatment arms (median 0.4 (IQR: 0.63–0.16) for BNPB; 0.4 (IQR: 0.8–0.2) for ER with aBT and 0.5 (IQR: 0.8–0.2) for ER without aBT). Supplementary Table S1 describes the key baseline patient characteristics, tumor features, and treatment aspects based on the treatment modality.

**Table 2:** Univariable analysis demonstrating key parameters in each treatment modalities

Parameter	ER with aBT vs ER without aBT	ER with aBT vs BNPB	ER without aBT vs BNPB
	OR (95%-CI) or CC *r*; *p*-value		
Age	*r* = 0.108	*r* = 0.230	*r* = 0.294
	*p* = 0.135	***p* < 0.0001**	***p* < 0.0001**
Age < 65 years old	0.64 (0.32–1.24)	0.41 (0.26–0.66)	0.26 (0.14–0.49)
	*p* = 0.193	***p* < 0.0001**	***p* < 0.0001**
Sex (females)	0.78 (0.43–1.41)	1.59 (1.01–2.50)	1.23 (0.71–2.15)
	*p* = 0.444	*p* = 0.054	*p* = 0.484
Extraocular extension	*	1.04 (1.01–1.07)	1.04 (1.01–1.07)
		*p* = 0.026	*p* = 0.206
LTT prior to the therapy	*r* = 0.098	*r* = − 0.141	*r* = − 0.022
	*p* = 0.174	***p* = 0.010**	*p* = 0.720
Largest basal tumor diameter	*r* = − 0.001	*r* = 0.449	*r* = 0.456
	*p* = 0.984	***p* < 0.0001**	***p* < 0.0001**
Posterior tumor margin:			
Parapapillary	0.69 (0.38–1.25)	0.32 (0.20–0.54)	0.22 (0.12–0.40)
	*p* = 0.226	***p* < 0.0001**	***p* < 0.0001**
Posterior to equator	0.97 (0.54–1.76)	0.87 (0.55–1.36)	0.84 (0.48–1.47)
	*p* = 1.000	*p* = 0.567	*p* = 0.570
Anterior to equator	2.60 (0.84–8.03)	4.28 (2.41–7.57)	11.12 (3.90–31.72)
	*p* = 0.100	***p* < 0.0001**	***p* < 0.0001**
Ciliary body involvement	1.90 (0.38–9.39)	24.00 (10.66–54.02)	45.50 (10.84–190.96)
	*p* = 0.721	***p* < 0.0001**	***p* < 0.0001**
Apex dose (Brachytherapy)	*r* = 0.857	*r* = -0.832	*r* = 0.775
	***p* < 0.0001**	***p* < 0.0001**	***p* < 0.0001**
Sclera contact dose (Brachytherapy)	*r* = 0.857	*r* = 0.815	*r* = 0.775
	***p* < 0.0001**	***p* < 0.0001**	***p* < 0.0001**
VA at diagnosis	*r* =− 0.031	*r* = − 0.039	*r* = − 0.064
	*p* = 0.680	*p* = 0.493	*p* = 0.305
VA < 0.05 decimal at diagnosis	1.47 (0.55–3.94)	0.63 (0.30–1.30)	0.92 (0.35–2.45)
	*p* = 0.634	*p* = 0.258	*p* = 0.806
VA < 0.05 decimal at 12 months	2.05 (0.97–4.34)	0.61 (0.36–1.05)	1.25 (0.61–2.58)
	*p* = 0.075	*p* = 0.093	*p* = 0.596
VA < 0.05 decimal at 36 months	1.60 (0.73–3.53)	1.47 (0.81–2.65)	2.35 (1.13–4.89)
	*p* = 0.327	*p* = 0.234	***p* = 0.024**
Enucleation at 36 months	3.15 (0.85–11.69)	0.59 (0.26–1.36)	1.86 (0.51–6.83)
	*p* = 0.100	*p* = 0.279	*p* = 0.562
Recurrence at 36 months	1.08 (0.17–6.75)	2.13 (0.57–7.93)	2.30 (0.49–10.83)
	*p* = 1.000	*p* = 0.384	*p* = 0.353

BNPB, Bi-nuclide plaque brachytherapy; ER, Endoresection; aBT, Adjuvant brachytherapy; OR, Odds ratio; CI, Confidence interval; CC, Correlation coefficient; VA, Visual acuity; RISN, Radiation-induced scleral necrosis; LTT, Largest tumor thickness

^*^This parameter could not be assessed, as it was not observed in any patient in either group; Bold values indicate statistically significant results (*p* <0.05)

### Primary study endpoints: VA at 12 and 36 months follow-up

**Fig. 2:**
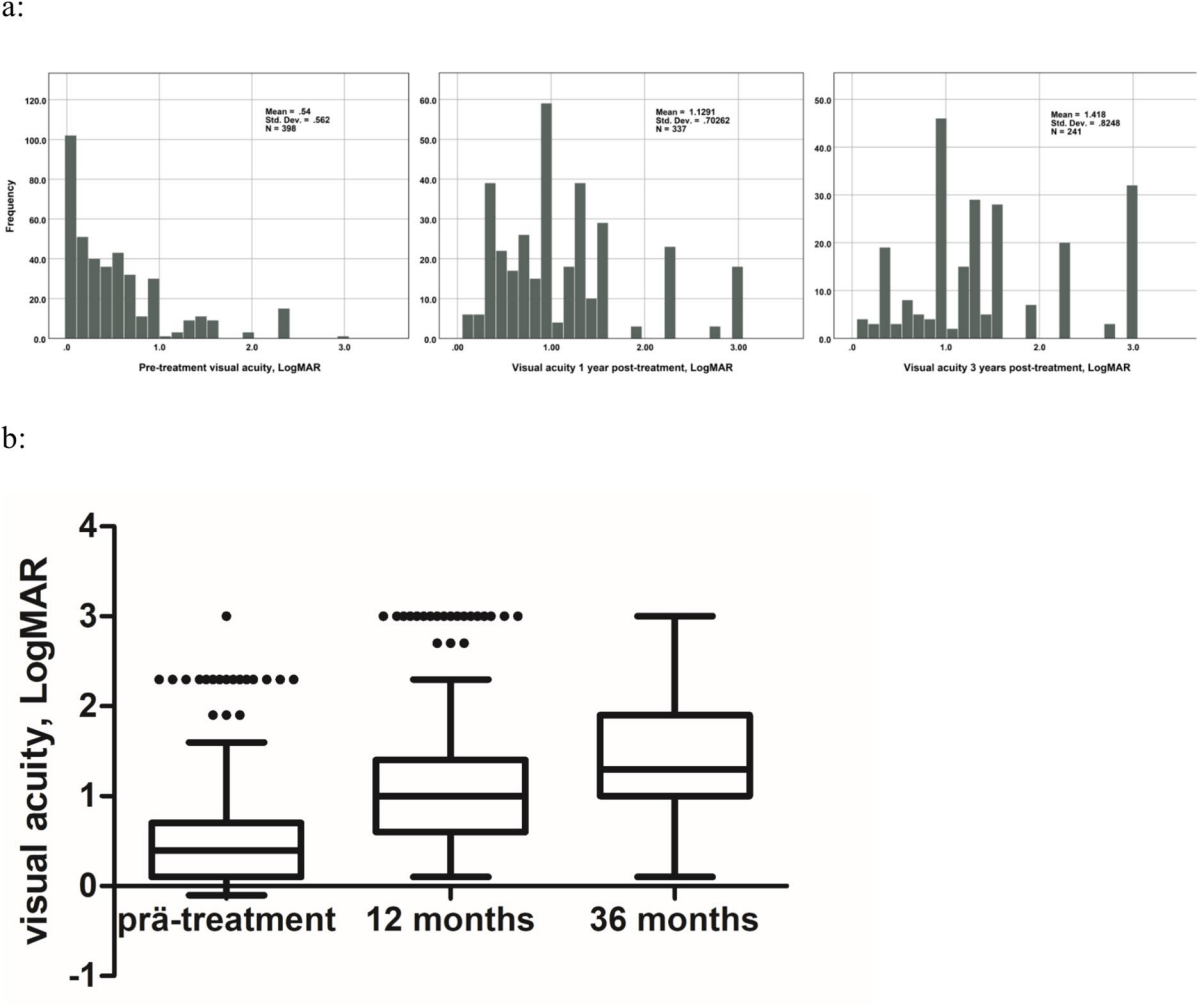
Distribution of visual acuity values in the entire cohort over the course of follow-up: pre-treatment visual acuity; visual acuity 12 and 36 months after therapy. LogMAR- logarithm of the minimum angular resolution

At 12 and 36 months, VA was available for 337 (84.7%) and 241 (60.6%) patients respectively. The median VA at 12 months was 0.1 decimal (IQR: 0.25–0.04) and LB was diagnosed in 86 (25.5%) cases. At 36 months, the median VA was 0.05 decimal (IQR: 0.1–0.01). 96 (39.8%) patients developed LB at this time interval. Figure 2 illustrates VA in the entire cohort at baseline, along with changes observed at 12 and 36 months following therapy.

In univariable analysis, ER without aBT was associated with lower risk to develop LB at 36 months as compared to BNPB (ER without aBT vs BNPB: *p* = 0.024; 27.1% vs 46.6%). A lower risk of LB after ER without aBT at 12 months (odds ratio [OR] = 0.30, *p* = 0.021) was confirmed in multivariable analysis (Table 3). Moreover, VA at diagnosis (per LogMAR increase, OR = 1.66, *p* = 0.030), LTT prior to the therapy (per mm increase, OR = 1.28, *p* = 0.010) as well as scleral contact dose (per Gy increase, OR = 1.00, *p* = 0.018) were also determined as significant risk factors for development of LB at 12 months. In turn, the patient’s age (per year increase, OR = 1.02, *p* = 0.037) and LTT prior the therapy (per mm increase, OR = 1.23, *p* = 0.046) were significantly associated with development of LB at 36 months after therapy.

**Table 3:** Multivariable logistic regression model with a backward stepwise conditional approach (the last steps are showed) analyzing development for LB at 12 and 36 months after treatment of large uveal melanoma (tumor thickness > 7 mm)

Parameter	aOR (95%–CI)	*p*-value
*Development of LB at 12 months*		
ER without aBT vs remaining cohort	0.30 (0.11–0.84)	**0.021**
Age, per year increase	1.02 (0.999–1.04)	0.058
Visual acuity at diagnosis, per LogMAR increase	1.66 (1.05–2.62)	**0.030**
LTT prior to the therapy, per mm increase	1.28 (1.06–1.54)	**0.010**
Scleral contact dose (Brachytherapy), per Gy increase	0.999 (0.998–1.00)	**0.018**
*Development of LB at 36 months*		
ER without aBT vs remaining cohort	0.61 (0.30–1.26)	0.181
Age, per year increase	1.02 (1.00–1.05)	**0.037**
LTT prior to the therapy, per mm increase	1.23 (1.00–1.51)	**0.046**

aOR, Adjusted odds ratio; CI, Confidence interval; ER, Endoresection; aBT, Adjuvant brachytherapy; LB, Legal blindness; LTT, Largest tumor thickness; LogMAR, Logarithm of the minimum angular resolution; Bold values indicate statistically significant results (*p* <0.05)

### Secondary study endpoints: tumor recurrence and secondary enucleation at 36 months

**Fig. 3:**
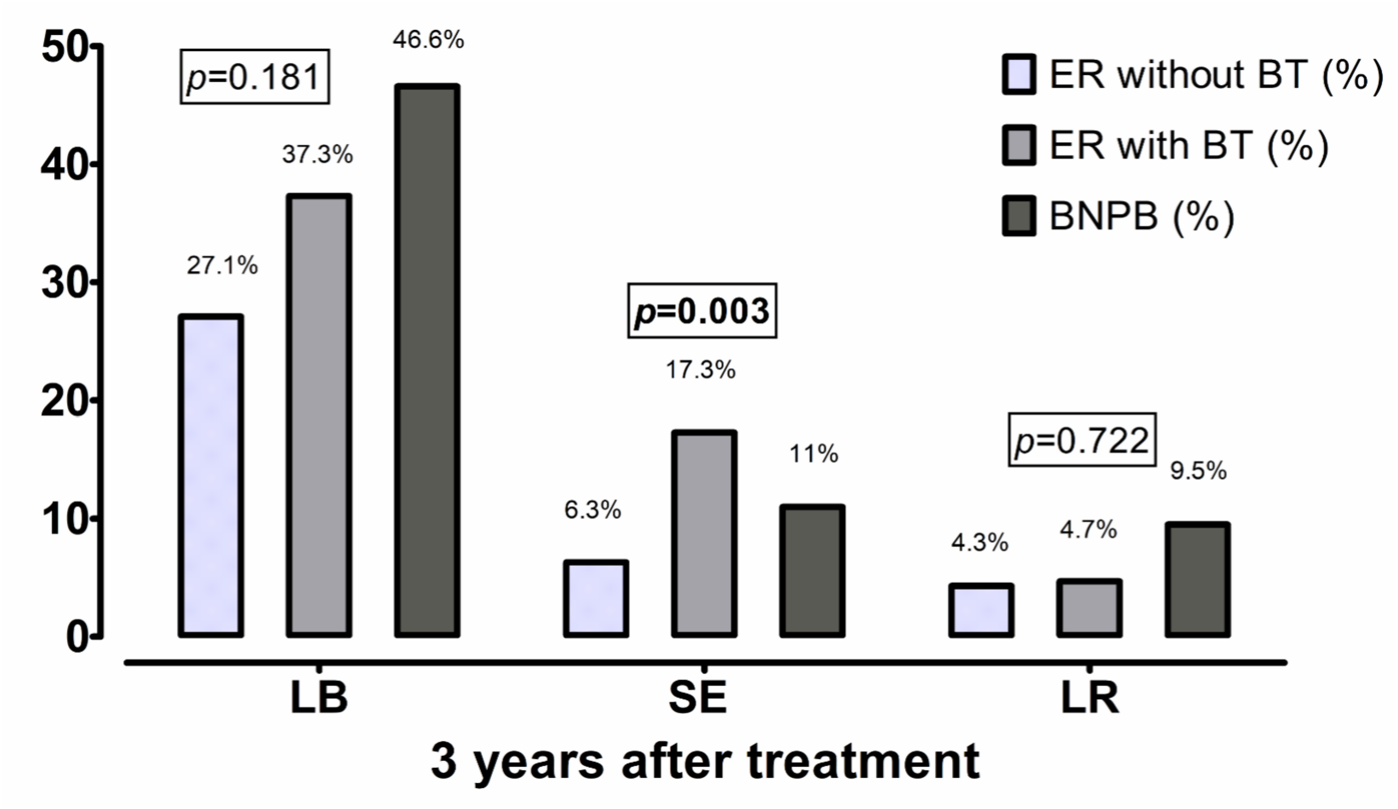
The proportion of patients with LB, SE and LR depending on the chosen treatment option at 36 months after therapy. LB-legal blindness; SE- secondary enucleation; LR-local recurrence; ER-endoresection; aBT-adjuvant brachytherapy; BNPB-bi-nuclide plaque brachytherapy; LogMAR- logarithm of the minimum angular resolution. Displayed *p*-values correspond to the comparison between ER without aBT subgroup vs remaining cohort

At 36 months follow-up, 12.0% (n = 29/241) of cases underwent SE. The most common indications to SE were local tumor failure (n = 12, 41.4%), followed by lack of the tumor control due to loss of visibility to the fundus (n = 6, 20.7%) and phthisis bulbi (n = 6, 20.7%), secondary glaucoma (n = 2, 6.9%), persistent retinal detachment (n = 1, 3.4%). In two cases (6.9%), SE was performed at the patient’s request due to a blind eye. Figure 3 illustrates the distribution of patients with LB, SE, and LR at 36 months.

Multivariable logistic regression analysis (online resource supplementary table S2) showed that patients’ age (per year increase, OR = 1.07, *p* = 0.003), LTT (per mm increase, OR = 1.54, *p* = 0.007), and scleral contact dose (per Gy increase, OR = 0.997, *p* = 0.002) were associated with SE at 36 months, whereas treatment modality (ER without aBT) was associated with a lower risk of SE (OR = 0.07, *p* = 0.003).

Information regarding LR was available for 225 patients (56.5%) at 36 months after therapy. LR was diagnosed in 16 (7.1%) of these cases. As mentioned above, 12 patients with LR underwent SE. Eye-preserving therapy was possible in four cases, consisting of transpapillary thermotherapy in three cases and ^106^Ru brachytherapy in one case. Multivariable logistic regression analysis (supplementary table S2) did not reveal independent predictors of LR among the recorded baseline parameters.

## Discussion

Both BNPB and ER are confirmed in our study as effective treatment options for large UM, enabling satisfactory eye and visual function preservation. Three years after therapy, eye preservation was achieved in 88.0% of cases and local tumor control was achieved in 92.9%. Individuals undergoing ER without aBT were at lower risk of LB at 12 months and SE at 36 months. Age and tumor thickness were identified as significant risk factors for LB and SE at 36 months.

Several studies have investigated the outcomes of eye-preserving therapy for large UMs [1, 2, 5, 15–20]. Research on brachytherapy with ^125^I for large UMs has reported SE rates of 16% and 24% at 5 years [1, 15]. Plaque radiotherapy for extra-large UMs (≥ 10 mm thickness) resulted in an eye preservation rate of 68% [20]. These results are comparable to reported rates of SE of up to 23% after ER [5, 6, 12, 21]. However, in two studies with a small patient sample, dealing with outcome after ER, eye retention was achieved even in 100% of cases over mean follow-up periods of 31 and 54.5 months, respectively [22, 23].

Despite initial concerns about potential tumor cell dissemination during ER, there was no difference in the number of circulating melanoma cells in peripheral blood following different therapeutic approaches, including ER [24, 25]. This could be attributed to the detection methods used or the sensitivity threshold of the assays. But studies comparing overall survival and disease-specific survival between ER and brachytherapy have found no significant differences, supporting the safety of this therapeutic option [12, 18].

In the same studies the authors also found no significant differences in eye retention rates between both groups. In contrast to these findings, our study showed the most favorable anatomical outcomes among the 3 therapeutic arms after ER without BT, with only 6.3% of patients requiring SE at 36 months. Multivariable regression analysis further confirmed this treatment modality as protective regarding SE at 36 months post-treatment.

A recent review evaluating proton beam therapy for UM of various sizes favored its use over brachytherapy, particularly for tumors larger than 8 mm, due to its unique physical properties that provide better sparing of critical ocular structures [26]. In this context, one study showed that patients with tumor height more than 7 mm who underwent ^125^I brachytherapy had significantly higher risk for enucleation than patients with lesions < 7 mm [27]. In contrast, a recent meta-analysis found no significant advantage of charged-particle therapy over brachytherapy in terms of SE rates [28]. Consistent with these findings, our cohort demonstrated a slightly higher eye retention rate of 88% following BNPB, compared to the 85.8% reported at 3 years post-treatment in a study evaluating long-term outcomes after proton therapy [29]. These results suggest that, with careful patient selection, plaque brachytherapy remains an effective treatment option for UM.Overall, 29 patients (12%) required SE within 36 months of treatment. In line with previous reports [30, 31], LR was the primary reason for SE in our study. At 36 months, 7.1% of patients developed LR. This outcome is comparable to LR rates reported following proton beam irradiation, where recurrence has been documented at approximately 5% [29]. Similarly, another study evaluating ^125^I brachytherapy in patients with large, American Joint Committee on Cancer T4-staged tumors reported a LR rate of 9% at the same follow-up interval [32]. Eye-preserving therapy of LR was possible in only a quarter of these cases in our cohort. Although the LR rate was lower in the ER-group, both with and without BT, compared to BNPB, the difference was not statistically significant. These findings are consistent with studies reporting no differences between brachytherapy and ER for LR rates [12, 18].

Comparing functional outcomes after therapy for UMs is challenging due to variations in patient cohorts, treatment modalities and, most importantly, differing endpoint cutoffs. As VA < 0.05 decimal has a significant impact on quality of life, making basic tasks such as reading or orientation nearly impossible, we chose this cutoff to identify individuals with the most severe visual impairment.

Notably, the literature specifically addressing functional outcomes following eye-preserving therapy for large UMs remains limited. However, it is well established that larger tumors are associated with greater visual loss compared to smaller and medium-sized tumors [33].

Findings from a small cohort suggest that ER may provide better visual outcomes than brachytherapy in selected cases [2]. However, another study comparing primary ER to primary ^125^I brachytherapy found no statistically significant difference in final VA between the two groups [18].

In the Collaborative Ocular Melanoma Study trial, which evaluated visual outcomes following brachytherapy with ^125^I, 76% of patients with large UM’s had a VA of ≤ 0.1 at 3 years [34]. Puusaari et al. reported a median VA of only light perception at 36 months following ^125^I brachytherapy for large UM, with only 4% of patients maintaining VA ≥ 0.1 decimal [1]. In contrast, our cohort demonstrated significantly better VA outcomes, with a median VA of 0.05 decimal at the same time point. 99 out of 241 patients (41%) in our study retained a VA of ≥ 0.1 decimal at 36 months post-treatment. In comparison, only 22.6% of patients treated with proton therapy achieved a VA of ≥ 0.1 decimal at the same follow-up interval [29]. Of course, we must acknowledge the potential selection bias due to missing follow-up data, as only 60.6% of patients were available at the 36 month evaluation. Incomplete follow-up data could potentially distort the interpretation of results. Nevertheless, the observed trends in visual outcome support the long-term efficacy of the evaluated treatment approaches.

The reported functional outcomes after ER are quite variable. In a study of Caminal et al., the Kaplan Meier probability of maintaining a VA equal or superior to 0.1 decimal at 5 years after ER was 59.9% [18]. Bechrakis et al. reported the median VA after ER with neoadjuvant proton radiotherapy of 0.1 decimal [21]. In contrast, another study evaluating outcome after ER with aBT revealed the median VA of only 0.01 decimal [6].

In our study, among the three treatment arms, the ER-group without aBT achieved the best results, with a median VA of 0.08 decimal at 36 months. At this interval 24 (50%) of 48 patients in this group remained VA of ≥ 0.1 decimal. But similarly to the results of Caminal et al. [18], the differences in VA in groups observed in our study at 36 months were not statistically significant. Consistently with other reports [14, 15, 32], patient’s age and tumor thickness were associated with VA deterioration with development of LB in multivariable analysis.

While the use of a unique bi-nuclide plaque specific to our institution may initially appear to limit the generalizability of our findings, conventional single nuclide ^125^I brachytherapy remains a widely accepted and suitable alternative. The dose exposure ratio between the bi-nuclide and the ^125^I plaques is approximately 0.7 and remains relatively stable with increasing distance from the plaque [4]. For tumors with a thickness of 10 mm or greater the ^125^I component becomes the predominant contributor [4]. Therefore, our findings are applicable to centers employing conventional ^125^I brachytherapy, especially in cases with tumor thickness exceeding 10 mm, where dosimetric differences between plaque types are minimal.

The eyes with large UM, which may have previously required enucleation, can now often be preserved through various therapeutic approaches, such as radiotherapy alone or in combination with surgical resection. Preserving functionality is another key goal of globe-conserving therapy, while VA is a complex outcome influenced by multiple factors.

Generally, an accurate therapy planning beginning with identification of proper treatment modality depending on tumor thickness and location, interdisciplinary cooperation of ocular oncologist and radiation therapist and physicist is crucial for therapy success.

## Study limitations

A retrospective design of our study is a limitation, as it relies on the accuracy of the documented parameters. Relatively high rate of cases lost to follow-up, particularly at 36 moths interval, also carries a substantial selection and information bias strongly restricting the statistical power of performed analysis. Bi-nuclide plaques are unfortunately no longer available but have been replaced in our institution by regular single nuclide ^125^I plaques. Although the dosimetric properties of BNPB and regular single nuclide iodine brachytherapy not the same, they are comparable and the herein described findings can be useful for indirect comparisons. Nevertheless, our study is based on one of the largest consecutive single-institutional cohorts and provides valuable long-term outcomes after eye preserving therapy of large UM.

## Conclusion

Both BNPB and ER are confirmed in our study as an effective treatment for large UM enabling the eye and function preservation. Among the investigated approaches, neoadjuvant irradiation with subsequent ER demonstrated the most favorable long-term outcomes, with a lower incidence of LB and SE.

## Supplementary Information

Below is the link to the electronic supplementary material.

Supplementary file1 (DOCX 19 KB)

## Data Availability

No datasets were generated or analysed during the current study.
