# Supplementary material for: Long-term outcome after treatment of large uveal melanoma
Source: Int Ophthalmol. 2025 Jul 8;45(1):279. doi: 10.1007/s10792-025-03624-0 (PMC12238149; doi:10.1007/s10792-025-03624-0)
Supplement: Supplementary file 1 — Supplementary file1 (DOCX 19 KB) [file 10792_2025_3624_MOESM1_ESM.docx]

**Supplementary Information**

**Table S1**. Key baseline patient characteristics, tumor features, and treatment aspects based on the treatment modality

| **Parameter** | **Number of cases (%) or  Median value (interquartile range)** | | |
| --- | --- | --- | --- |
|  | **BNPB** | **ER with aBT** | **ER without aBT** |
| Number of patients | 204 | 127 | 67 |
| Age, years | 66.5 (55.3-76.4) | 60.9 (50.5-67.5) | 55.3 (48.9-65.0) |
| Age < 65 years old | 93 (45.6%) | 85 (67.0%) | 51 (76.1%) |
| Sex (females) | 102 (50.0%) | 49 (38.6%) | 30 (44.8%) |
| Follow-up duration | 34.3 (14.4-49.9) | 32.5 (9.3-62.8) | 40.9 (27.1-46.9) |
| TNM T2a  T2b  T3a  T3b  T3c  T3d  T4a  T4b | 9  15  73  84  3  5  4  11 | 34  0  75  6  0  0  11  1 | 20  0  42  2  0  0  3  0 |
| Apex dose (Brachytherapy) | 72.0 (70.2-83.5) | 142.0 (133.6-154.0) | 0 |
| Sclera contact dose (Brachytherapy) | 895.7 (780.1-1054.0) | 432.3 (407.4-464.3) | 0 |
| Extraocular extension | 8 (3.9%) | 0 (0%) | 0 (0%) |
| Ciliary body involvement | 119 (58.3%) | 7 (5.5%) | 2 (3.0%) |
| LTT prior to the therapy, mm | 8.6 (7.9-9.7) | 9.2 (8.1-10.3) | 8.7 (7.8-9.8) |
| Largest basal tumor diameter | 15.0 (13.2-16.3) | 11.9 (10.0-13.5) | 11.8 (10.4-13.4) |
| Posterior tumor margin† :  parapapillary  posterior to equator  anterior to equator | 36 (17.7%)  82 (40.6%)  84 (41.4%) | 50 (40%)  56 (44.1%)  18 (14.2%) | 33 (49.3%)  30 (44.8%)  4 (6.0%) |
| VA at diagnosis, decimal | 0.4 (0.63-0.16) | 0.4 (0.8-0.2) | 0.5 (0.8-0.2) |
| VA at 12 months, decimal | 0.1 (0.25-0.05) | 0.06 (0.2-0.03) | 0.125 (0.32-0.05) |
| VA at 36 months, decimal | 0.05 (0.1-HM) | 0.06 (0.1-0.01) | 0.08 (0.32-0.03) |
| Patients with LB at diagnosis | 17 (8.3%) | 16 (12.6%) | 6 (9%) |
| Patients with LB at 12 months | 39 (23.1%) | 35 (33.0%) | 12 (19.4%) |
| Patients with LB at 36 months | 55 (46.6%) | 28 (37.3%) | 13 (27.1%) |
| Enucleation at 36 months | 13 (11.0%) | 13 (17.3%) | 3 (6.3%) |
| Recurrence at 36 months | 11 (9.5%) | 3 (4.7%) | 2 (4.3%) |

Abbreviations: BNPB- bi-nuclide plaque brachytherapy; ER-endoresection; aBT- adjuvant brachytherapy; VA-visual acuity; HM-hand motion; LB-legal blindness; LTT-largest tumor thickness

*- patients who underwent enucleation were categorized as lost to follow-up; †- in 5 cases posterior tumor margin was not documented.

**Table S2.** Multivariable logistic regression model with a backward stepwise conditional approach (the last steps are showed) analyzing secondary enucleation and occurrence of recurrence at 36 months after treatment of large uveal melanoma (tumor thickness > 7 mm)

| **Parameter** | **aOR (95%-CI)** | ***p*-value** |
| --- | --- | --- |
| ***Secondary enucleation at 36 months*** | | |
| ER without aBT vs remaining cohort | 0.07 (0.01-0.41) | **0.003** |
| Age, per year increase | 1.07 (1.02- 1.11) | **0.003** |
| LTT prior to the therapy, per mm increase | 1.54 (1.12-2.12) | **0.007** |
| Scleral contact dose, per-Gy-increase | 0.997 (0.995-0.999) | **0.002** |
| ***Recurrence at 36 months*** | | |
| ER without aBT vs remaining cohort | 0.75 (0.16-3.61) | 0.722 |
| Extraocular extension | 8.28 (0.68-100.14) | 0.097 |

**Abbreviations**: aOR-adjusted odds ratio; CI- Confidence interval; ER-endoresection; aBT-adjuvant brachytherapy; LTT-largest tumor thickness
